# Transcutaneous injection of triamcinolone acetonide for persistent glottic granulation after laser microsurgery

**DOI:** 10.1016/j.bjorl.2023.01.006

**Published:** 2023-02-09

**Authors:** Ying Zhang, Shisheng Li

**Affiliations:** Central South University, The Second Xiangya Hospital, Department of Otolaryngology, Head and Neck Surgery, Hunan, China

**Keywords:** Early glottic cancer, transoral laser microsurgery, Fibrolaryngoscopy, Persistent granuloma, Triamcinolone acetonide, Transcutaneous intralesional injection

## Abstract

**Objective:**

The aim of study was to demonstrate that transcutaneous intralesional injection of Triamcinolone Acetonide (TA) under fibrolaryngoscopy could be an option for persistent granulation after Transoral Laser Microsurgery (TLM) in glottic cancer patients.

**Methods:**

We recruited 32 patients, who had conservative treatment but failed. 20 patients accepted TA injection monthly until the granulation disappeared or did not shrink further. 12 patients chose to closely monitor.

**Results:**

For the 20 patients, 17 (85.0%) patients’ granulations completely disappeared. 3 (15.0%) patients’ granulations had reduced 80%. For the 12 patients, 3 (25.0%) patients’ granulations disappeared but 9 (75%) patients’ granulations did not have an obvious change. Recurrence was not observed.

**Conclusion:**

Our experience showed that transcutaneous intralesional TA injection for persistent granulation after TLM through cricothyroid membrane is an efficient, security, harmless and low recurrence method. Especially suitable for huge granulation which blocks the glottis and recur after a second operation.

## Highlights

• Persistent granulation could not disappear after conservative treatment.
• Reoperation was not recommended.
• Triamcinolone Acetonide (TA) injection in vocal fold is mature.
• TA could accelerate the disappear of granulation or persistent granulation.
• Suitable for huge granulation which blocks the glottis and the recur.

## Introduction

Radiotherapy (RT), Transoral Laser Microsurgery (TLM) and open surgery are current comment treatment options for early glottic cancer.^1^, ^2^, ^3^ With the advantages of lower morbidity, improved organ and function preservation, ease of administration, the potential to be repeated and the ability to keep open laryngeal surgery and RT available, TLM is increasingly being a major modality in the management of early glottic cancer, even for T3 glottic.^4^, ^5^, ^6^, ^7^

Granulation was one of the common complications of TLM.^5^, ^8^ It is the mucosal growth of predominantly granulation tissue that occurs as a response to tissue injury and irritation, usually occur 1–2 months after TLM in more than 50% of patients and could disappear spontaneously in 6 months in nearly half patients.^7^, ^8^ As a result, it will be replaced by the scar and then stable mucosa can be formed.^9^ Persistent granulation, which exists more than 6 months after TLM makes it difficult to distinguish between normal healing and tumor recurrence, increasing the psychological pressure of patients. It has a bad effect on the recovery of voice and influences the observation of wounds under the laryngoscope. Large granulation can affect breathing and even cause laryngeal obstruction. Previous reports have shown that granulation persistence over time was may significantly associated with diabetes mellitus, thyroid cartilage exposure and affected surgical margins.^8^

There is no consensus on how granulation should be handled after TLM. Primary conservative management includes the zinc drug and proton pump inhibitor. In patients with persistent granulation, the failure of conservative treatment may be related to poor compliance. Because delayed healing compared to ‘cold’ surgical techniques, wait and see policy was believed to be necessary for up to three months to avoid unnecessary biopsy was necessary.^10^ When granulation exists for more than 6 months after TLM, secondary surgery or close monitor could be chosen.^5^, ^11^ It was reported that in the potential routine revision surgery, the probability of negative histopathology would have been higher than 70%, although the presence of granulation tissue at month 6 was not systematically related to tumor relapse, revision surgery was strongly recommended.^8^ The low positive rate of histopathology, second damage, and inconvenience of general anesthesia are barriers to choosing a second operation reoperation.^5^, ^12^ But close monitoring leads patients feeling uncomfortable both psychologically and physically, which is a struggle for the patient. In addition, the bad effect on the voice will exist all the time.

Triamcinolone Acetonide (TA) vocal fold injection has been reported for the treatment of benign laryngeal lesions, such as vocal fold nodule and contact granuloma. Sang-Hyuk Lee and Wang, CT had reported that local injection of TA was a useful and safe treatment option for vocal nodules and refractory vocal process granuloma.^13^, ^14^

Based on previous research, the purpose of this study was to demonstrate that transcutaneous intralesional TA injection under fibrolaryngoscopy could be a potential treatment option for persistent granulation after TLM in glottic cancer patients. Especially suitable for huge granulation which blocks the glottis and recur after a second operation.

## Methods

### Patients

From October 2016 to February 2019 in The Second Xiangya Hospital, there were 90 glottic cancer patients who presented granulation after TLM. After 6 months of conservative management that includes zinc drug, proton pump inhibitor and atomization treatment. The granulation of 55 (61.1%) patients disappeared and the remaining 35 patients had failed. The 35 patients all underwent a biopsy. 3 patients were malignant and then underwent reoperation. The other 32 patients had negative histopathology. Pathological evidence of the lesion was available in all 35 patients (the tissue was achieved with a flexible laryngoscope). In 32 patients, the age distribution ranged from 45 to 67 years, with a mean age of 56.0 years. 31 males and 1 female. The patients with T1N0M0 and T2N0M0 patients accepted type III TLM. All 32 patients have unilateral glottic carcinoma and surgical margins were negative. 4 patients who accepted type III TLM exposed the thyroid cartilage during the operation. Of the 32 patients, 20 patients consented to the injection treatment and were assigned to injection group. They were treated TA injection monthly. 12 patients rejected injection and continued closely monitored. They were assigned to monitoring group. The protocol of treatment has been approved by the Institutional Review Board and the doctors have obtained written informed consent from each participant. No patient received any other surgical treatment prior to injection of TA. The information of the patients is given in Table 1.

**Table 1:** Clinical data of 32 male unilateral glottic carcinoma patients with laryngology persistent granulation following TLM and the surgical margins were all negative.

Case	Age (y)	TNM	Laryngeal endoscopic cordectomy	Diabetes	Exposure of the thyroid cartilage
1	51	T1N0M0	Type III	No	No
2	59	T2N0M0	Type III	No	No
3	45	T1N0M0	Type III	No	No
4	50	T1N0M0	Type III	No	No
5	62	T3N0M0	Type Va	No	Yes
6	54	T3N0M0	Type Va	Yes	Yes
7	48	T1N0M0	Type III	No	No
8	50	T2N0M0	Type III	No	No
9	49	T1N0M0	Type III	No	No
10	55	T2N0M0	Type III	No	No
11	56	T1N0M0	Type III	No	No
12	62	T1N0M0	Type III	No	No
13	60	T3N0M0	Type Va	No	Yes
14	47	T1N0M0	Type III	No	No
15	54	T2N0M0	Type III	No	No
16	58	T1N0M0	Type III	Yes	No
17	63	T2N0M0	Type III	No	No
18	56	T1N0M0	Type III	No	No
19	64	T1N0M0	Type III	Yes	No
20	59	T2N0M0	Type III	Yes	No
21	50	T2N0M0	Type III	No	No
22	70	T1N0M0	Type III	No	No
23	67	T2N0M0	Type III	No	No
24	55	T1N0M0	Type III	No	No
25	56	T2N0M0	Type Va	Yes	Yes
26	60	T1N0M0	Type III	No	No
27	60	T2N0M0	Type III	No	No
28	62	T3N0M0	Type Va	No	Yes
29	50	T1N0M0	Type III	No	No
30	54	T1N0M0	Type III	No	No
31	49	T1N0M0	Type III	No	No
32	58	T1N0M0	Type III	No	No

### TA injection procedure and follow-up

**Figure 1:**
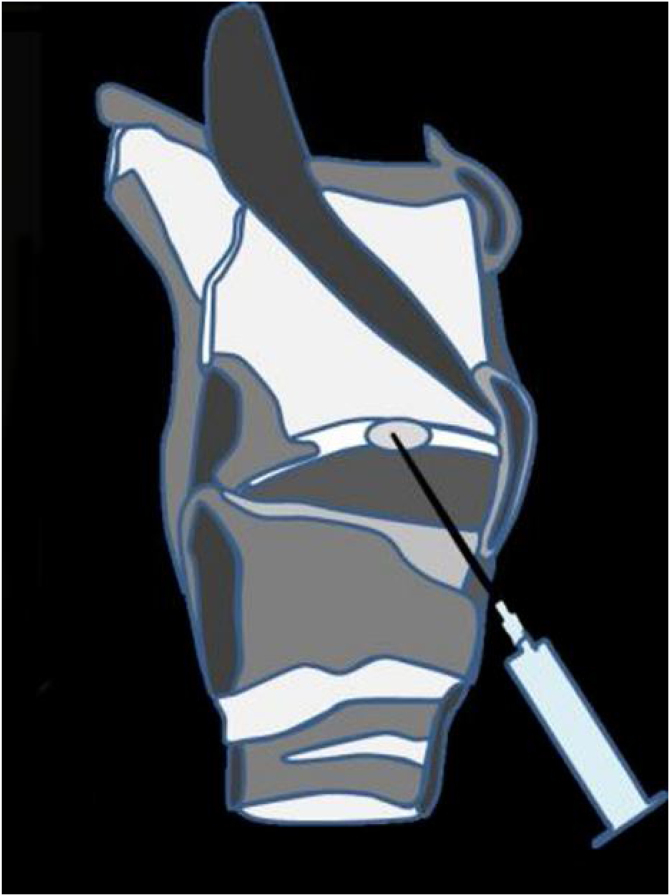
Schematic diagram of the injection process.

The TA injection was performed under local anesthesia of the pharynx and larynx, including spraying 10% xylocaine over the pharynx, tonsil, vallecula and epiglottis. The patient’s nasal cavity was topicalized with a 50/50 mixture of 0.05% oxymetazoline and 10% xylocaine. The subcutaneous tissue that overlies the cricothyroid membrane was injected with approximately 0.5 mL of lidocaine. The flexible laryngoscope was then passed through the patient’s nasal cavity, opposite the granulation to be injected. The needle advanced through the cricothyroid membrane into the subglottic airway as close to the granulation as possible. The injected dose was 5 mg (0.5 mL) TA and then the needle was pulled out. Fig. 1 is the schematic diagram of injection. After injection, the patient observed at least half an hour in the outpatient clinic. Patients were asked for regularly followed up 6 months after the last injection. Clinical examinations, including an indirect mirror examination of the larynx with or without a laryngoscope, were performed during subsequent follow-up.

## Results

**Figure 2:**
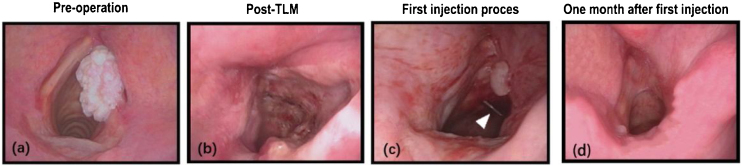
(a) A 54-year-old man with right glottic cancer. (b) Laryngoscopy on the second day after surgery. (c) First injection process and the white arrow points to the needle. (d) One month after injection, the granulation completely disappeared.

**Figure 3:**
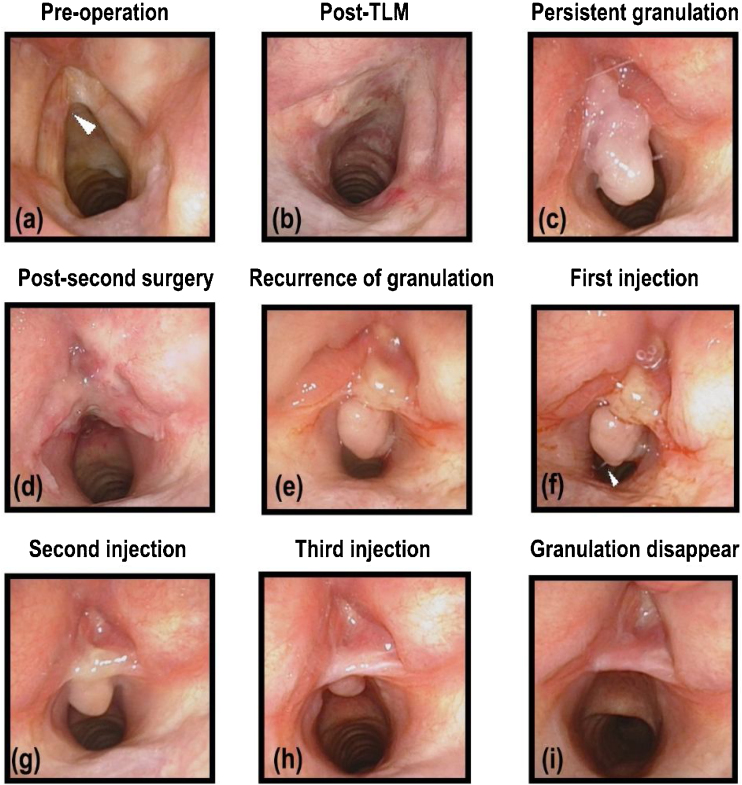
(a) A 54-year-old woman with a left malignant lesion, who underwent laser surgery 2 years ago because of the right glottic cancer. The white arrow points to the lesion (b) Laryngoscopy on the second day after the first surgery. (c) Persistent granulation after the first surgery. (d) Laryngoscopy on the second day after the second surgery. (e) One week after the second surgery, the granulation reappeared. (f) In the process of the first injection, the white arrow points to the needle. (g) Second injection. (h) Third injection. (i) One month after the third injection, the granulation disappeared completely.

After 1–3 courses of steroid injections, the injection group revealed subjective and objective improvements. 17 (85.0%) patients’ granulations completely disappeared. The granulation size of the remaining 3 (15.0%) patients had reduced by 80% after the first injection but did not shrink further in the next 2 injection courses. 3 patients received 1 injection course, 13 patients received 2 injection course, 4 patients received 3 injection course and the mean was 2.05 ± 0.622 SD. Fig. 2 shows a 54-year-old man with a persistent granuloma on the right side. After 1 course of transcutaneous intralesional injection of Triamcinolone Acetonide (TA) under fibrolaryngoscopy, the granulation had completely disappeared. Fig. 3 shows a 54-year-old woman with a left malignant lesion, who underwent laser surgery 2 years ago because of right glottic cancer. After TLM, the granulation did not disappear after 6 months. Since the granulation block part of the glottis, she has mild throat obstruction and accepted a second surgery. The pathology in the second surgery was benign. It is worth mentioning that 1 week after the second surgery the granulation reappears again and again causing throat obstruction. Since the second surgery was useless, she accepted the injection treatment. After 3 rounds of injection, the granulation completely disappeared. The most common side effects after intralesional steroid injections were an itchy throat and cough, which appeared in 12 patients and resolved spontaneously within 1 week after injection. In the monitoring group, the granulation of 3 (25.0%) patients disappeared and there was no obvious change in 9 (75.0%) patients. During the 6-month follow-up period, recurrence granulation was not observed in cured patients. The shrinkage of the granulation volume is given in Table 2, Table 3. The Level of Evidence is IIb.

**Table 2:** Treatment outcomes of transcutaneous intralesional TA injections.

Case	Reduction rate (%)		
	1 (m)	2 (m)	3 (m)
1	50	80	100
2	80	100	‒
3	100	‒	‒
4	80	100	‒
5	80	80	80
6	80	100	‒
7	80	80	100
8	80	100	‒
9	80	100	‒
10	50	100	‒
11	100	‒	‒
12	80	80	100
13	80	80	80
14	80	100	‒
15	50	100	‒
16	80	80	80
17	50	100	‒
18	100	‒	‒
19	80	100	‒
20	50	100	‒

**Table 3:** Outcomes of monitor group.

Case	Reduction rate (%)		
	1 (m)	2 (m)	3 (m)
1	0	0	0
2	0	0	0
3	50	80	100
4	0	0	0
5	0	0	0
6	0	0	0
7	50	80	100
8	0	0	0
9	0	0	0
10	80	100	‒
11	0	0	0
12	0	0	0

## Discussion

In-office laryngeal injection techniques have been reported for paralysis of the vocal cord paralysis, laryngeal contact granuloma and has been continuously modified and improved to be a safe and effective method.^14^, ^15^, ^16^, ^17^ There are three commonly used transcutaneous approaches: transcricothyroid, transcartilagenous and transthyrohvoid.^3^ We chose transcricothyroid approach because most of the granulation was located in the glottic or subglottic area, which was relatively easy to reach through transcricothyroid approach. In addition, there are no important artery and nerve in this trajectory. Although intralesional steroid injections for vocal process granuloma have been reported using a 70-degree rigid laryngoscope in transoral approach.^14^ It is very easy to induce a pharyngeal reflex through transoral approach which will increase the difficulty of operation and affect the precision of the injection. Furthermore, the patient must hold his tongue by himself during the injection process which is painful and uncooperative.^18^, ^19^, ^20^ In our cases, patients had good tolerance to the procedure and were pleased to accept this treatment because of the lower cost and less suffering.

TA was useful in the treatment of many granulation diseases, such as Central Giant Cell Granulation (CGCG), facial granulation, and pyogenic granulation and was shown to be safe and effective in the injection approach in the form of an aqueous suspension.^21^, ^22^, ^23^, ^24^ Topical steroid injection has the advantages of higher concentration at the primary site and lower rates of adverse effects (opportunistic fungal infection and vocal fold atrophy).^14^, ^25^ As a long-acting synthetic corticosteroid, TA had the advantages of long injection cycle, being well absorbed and not having the possibility of allergic reaction. TA has also been reported as a recommended treatment to reduce central foveal thickness and improve visual in patients with uveal melanoma with radiation maculopathy after proton beam therapy.^26^ Research shows that TA injection therapy after esophageal cancer surgery and combined with ED (endoscopic dilation) are effective and safe in the management of stenosis.^27^ At present, there are no relevant articles on the evaluation of systemic influence on cancer patients regarding the intralesional using of TA. However, no increase in tumor recurrence rate caused by TA has been reported in current reports. The patients in our injection group were not observed tumor recurrence in followed up 6 months. Since there are some reports of severe infection or necrosis cases following local steroid injection after transoral surgery for radiation failure,^28^ steroid injections are not recommended in these cases.

**Figure 4:**
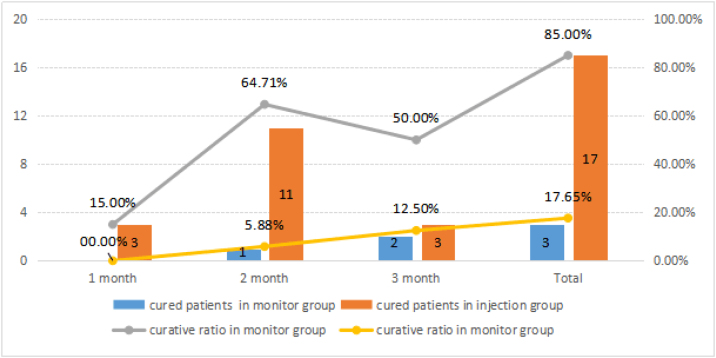
Comparison of the curative ratio in the monitor group and the injection group and the time variation.

In our study, we found the potential role of transcutaneous intralesional TA injection for persistent granulation after TLM. Intralesional TA injection through the cricothyroid membrane is performed safely with a high cure rate. In the injection group, the cured rate was 85.0% (17/20) and during the 6 months follow-up period, recurrence was not observed. In the monitor group, 3 (25.0%) patients’ granulations disappeared and there was no obvious change in 9 (75.0%) patients. The cure rate of the injection group was significantly higher than that of the monitor group. Furthermore, there were 3 cured patients in the first month of the injection group, but no patient was cured in that of the monitor group and the main cured patients distributed over the second month in the injection group compared the main cured patients distributed over the third month in the monitor group, indicating that injection treatment could also promote recovery time (Fig. 4). Although we have fewer cases, the outcome preliminaryly shows that intralesional TA injection under fibrolaryngoscopy was an effective method. We recommend that the 3 patients whose granulation did not disappear after 3 months of injection treatment continue to be closely monitored, since they already had biopsy 6 months after surgery.

Recurrence is the major defect of the second surgery. Since granulation occurred as an inflammatory reaction in response to mechanical or functional trauma, the wound caused by the by the second surgery may cause recurrence. As the case in Fig. 3, one week after the second surgery, the granulation appeared again and caused laryngeal obstruction again, after 3 injection courses, the granulation disappeared completely. During the 6-month follow-up period, no recurrence was observed in our cured patients. Probably because instead of an open wound on the mucosa, TA injection technique could minimize trauma and inhibit granulation regeneration.

Our study had several limitations. First, more patients and a longer follow-up are needed in our study. Moreover, we did not obtain the change in the voice data after treatment, which we hope will be done in a later study. At present, there are no relevant articles referring to the evaluation of systemic influence in cancer patients on intralesional use of TA and there is no reported increase in tumor recurrence rate caused by TA injection. It has little probability of puncturing the anterior jugular vein in the injection process. Further studies of this treatment are necessary to confirm the recurrence of granulation and the systemic and local influence in cancer patients after intralesional use of TA.

## Conclusions

Transcutaneous intralesional TA injection to persistent granulation after TLM for patients with glottic cancer through the cricothyroid membrane under the guidance of fibrolaryngoscope is an efficient, secure, harmless, and low recurrence technique. We recommend it could be as a treatment method for persistent granulation after TLM. Especially suitable for huge granulation which blocks the glottis and the recur after a second operation.

## Funding

10.13039/501100001809National Natural Science Foundation of China (81402502) supported this research.

## Ethical approval

All procedures performed in studies involving human participants were in accordance with the ethical standards of the institutional and/or national research committee and with the 1964 Helsinki declaration and its later amendments or comparable ethical standards.

## Conflicts of interest

The authors declare no conflicts of interest.
